# A forward genetic screen reveals a primary role for *Plasmodium falciparum* Reticulocyte Binding Protein Homologue 2a and 2b in determining alternative erythrocyte invasion pathways

**DOI:** 10.1371/journal.ppat.1007436

**Published:** 2018-11-29

**Authors:** Susana Campino, Alejandro Marin-Menendez, Alison Kemp, Nadia Cross, Laura Drought, Thomas D. Otto, Ernest Diez Benavente, Matt Ravenhall, Frank Schwach, Gareth Girling, Magnus Manske, Michel Theron, Kelda Gould, Eleanor Drury, Taane G. Clark, Dominic P. Kwiatkowski, Alena Pance, Julian C. Rayner

**Affiliations:** 1 Malaria Programme, Wellcome Sanger Institute, Wellcome Genome Campus, Hinxton, Cambridge, United Kingdom; 2 Faculty of Infectious and Tropical Diseases, London School of Hygiene and Tropical Medicine, London, United Kingdom; 3 Centre of Immunobiology, Institute of Infection, Immunity & Inflammation, College of Medical, Veterinary and Life Sciences, University of Glasgow, Glasgow, United Kingdom; 4 Faculty of Epidemiology and Population Health, London School of Hygiene and Tropical Medicine, London, United Kingdom; 5 Wellcome Trust Centre for Human Genetics, University of Oxford, Oxford, United Kingdom; National Institutes of Health, UNITED STATES

## Abstract

Invasion of human erythrocytes is essential for *Plasmodium falciparum* parasite survival and pathogenesis, and is also a complex phenotype. While some later steps in invasion appear to be invariant and essential, the earlier steps of recognition are controlled by a series of redundant, and only partially understood, receptor-ligand interactions. Reverse genetic analysis of laboratory adapted strains has identified multiple genes that when deleted can alter invasion, but how the relative contributions of each gene translate to the phenotypes of clinical isolates is far from clear. We used a forward genetic approach to identify genes responsible for variable erythrocyte invasion by phenotyping the parents and progeny of previously generated experimental genetic crosses. Linkage analysis using whole genome sequencing data revealed a single major locus was responsible for the majority of phenotypic variation in two invasion pathways. This locus contained the *PfRh2a* and *PfRh2b* genes, members of one of the major invasion ligand gene families, but not widely thought to play such a prominent role in specifying invasion phenotypes. Variation in invasion pathways was linked to significant differences in *PfRh2a* and *PfRh2b* expression between parasite lines, and their role in specifying alternative invasion was confirmed by CRISPR-Cas9-mediated genome editing. Expansion of the analysis to a large set of clinical *P*. *falciparum* isolates revealed common deletions, suggesting that variation at this locus is a major cause of invasion phenotypic variation in the endemic setting. This work has implications for blood-stage vaccine development and will help inform the design and location of future large-scale studies of invasion in clinical isolates.

## Author summary

*Plasmodium* parasites cause more than 200 million cases of malaria each year. All the symptoms of malaria are caused after *Plasmodium* parasites invade human red blood cells. Once inside, they grow, multiply and break open the red blood cells to release new parasites. This cycle is repeated every 48 hours, rapidly amplifying the number of parasites and causing severe anemia and other complications. *Plasmodium falciparum*, the parasite species responsible for almost all malaria deaths, can use multiple different pathways to invade human red blood cells, but the relative importance of each is not well understood. We tested the invasion pathways used by a collection of closely related parasites and compared their genome sequences to identify the genes responsible. This analysis revealed that expression differences in two neighboring genes of the Reticulocyte Binding Homologue family are responsible for most of the variation in two invasion pathways. *P*. *falciparum* may use variation in these genes to avoid the immune system or adapt to specific blood groups, which has important implications for vaccine development against malaria.

## Introduction

*Plasmodium falciparum* is an obligate intracellular parasite, unable to replicate outside a host cell. During the blood stages of its complex life cycle, *P*. *falciparum* parasites must transition from one erythrocyte to another. The process by which *P*. *falciparum* merozoites recognize and invade human erythrocytes is therefore critical for parasite survival, but also represents a brief window when it is extracellular and vulnerable to the host immune system. To guarantee its replication, the parasite has evolved a series of strategies to evade the host immune response during erythrocyte invasion. These strategies include using multiple alternative pathways to recognize erythrocytes, which are thought to allow the parasite population to survive if a specific invasion route is blocked by the immune response, or to adapt to different human erythrocyte surface polymorphisms.

These alternate invasion pathways, and the receptor-ligand interactions that specify them, have been the subject of intensive research. Studies have employed *in vitro* invasion assays, inhibitory antibodies targeting specific ligands, enzyme treated or genetically deficient erythrocytes lacking specific receptors, and *P*. *falciparum* lines that have been genetically manipulated to delete specific ligands (reviewed in [1]). Together, these studies have suggested that alternative invasion is largely specified by members of the *P*. *falciparum* Erythrocyte Binding Antigen (PfEBA) and Reticulocyte binding Homologs (PfRh) multi-gene families (reviewed in [2]). Erythrocyte receptors have been identified for several PfEBAs and PfRhs, including three members of the Glycophorin family that are receptors for PfEBAs [3–5], and Complement Receptor 1 (CD35) that acts as a receptor for PfRh4 [6, 7]. However, the *P*. *falciparum* genes encoding these ligands can each be deleted without compromising parasite viability, implying that these receptor-ligand interactions are not absolutely required for invasion to occur [4, 8, 9]. By contrast, other receptor-ligand interactions, specifically those between PfRh5 and its receptor Basigin [10], and between AMA1 and RON2 [11], do appear to be essential. This, coupled with detailed video microscopy studies, has led to a model where the receptor-ligand interactions that are redundant operate at a relatively early stage during invasion [12], and perhaps provide the parasite with alternative pathways by which to invade, while those that are essential operate at later stages that are absolutely required in all strains [13].

While this picture of alternate invasion pathways involving PfRh and PfEBA ligand families is well established, it is not at all clear what molecular mechanisms underpin the phenomenon. In one case, a *P*. *falciparum* strain can be made to switch between invasion pathways under selection pressure or when exposed to gentle shaking, and this switch is accompanied by transcriptional up-regulation of PfRh4 [14–16]. However, this switching phenomenon is far from universal among lab-adapted isolates [15] and whether it occurs in clinical isolates is not known. Invasion phenotyping studies with *P*. *falciparum* clinical isolates from Africa, Asia and South America show that variation in invasion pathways, as measured by sensitivity to enzyme treatment, is universal (reviewed in [17]). However, although some of these studies have linked alternative invasion pathways to variation in the sequence or expression of specific PfRhs or PfEBAs [18–23], the relative contribution of each ligand to a given pathway is not clear; it is likely that most such pathways are controlled by multiple genes.

Reverse genetics, deleting candidate genes one at time, has been used to great effect in dissecting the relative contribution of individual PfRh and PfEBA ligands (reviewed in [1]). However, this approach by definition only considers known candidate genes. In other eukaryotic systems genetic variants affecting complex phenotypes have been identified using forward genetic approaches such as quantitative trait loci (QTL) mapping. In such approaches, two strains of known phenotype are crossed, and the phenotype and genotype of the parents and progeny are compared to identify QTLs associated with a given phenotype. Due to technical challenges and ethical considerations, only a limited number of experimental genetic crosses have been carried out in *P*. *falciparum* [24–26], although the recent development of humanized mouse models may make such crosses more routine [27]. *P*. *falciparum* genetic crosses have previously been used to identify genes responsible for drug resistance [26, 28, 29] and primate host tropism [25]. We used the parents and progeny of two experimental crosses, 7G8 (Brazil) x GB4 (Ghana) and HB3 (Honduras) x Dd2 (Laos) in order to take an unbiased approach to identify genes underpinning alternative invasion pathways. Detailed phenotyping using a two-colour flow cytometry assay to quantitate alternative invasion [30] was combined with whole genome sequencing data [31] to perform QTL analysis. Despite the presumed complexity of invasion phenotypes, in the 7G8xGB4 cross a single major locus was identified as responsible for the majority of variation in two of the most variable alternative invasion pathways.

## Results

### Erythrocyte invasion phenotypes of parents and progeny from the 7G8xGB4 *P*. *falciparum* experimental genetic cross

**Fig 1:**
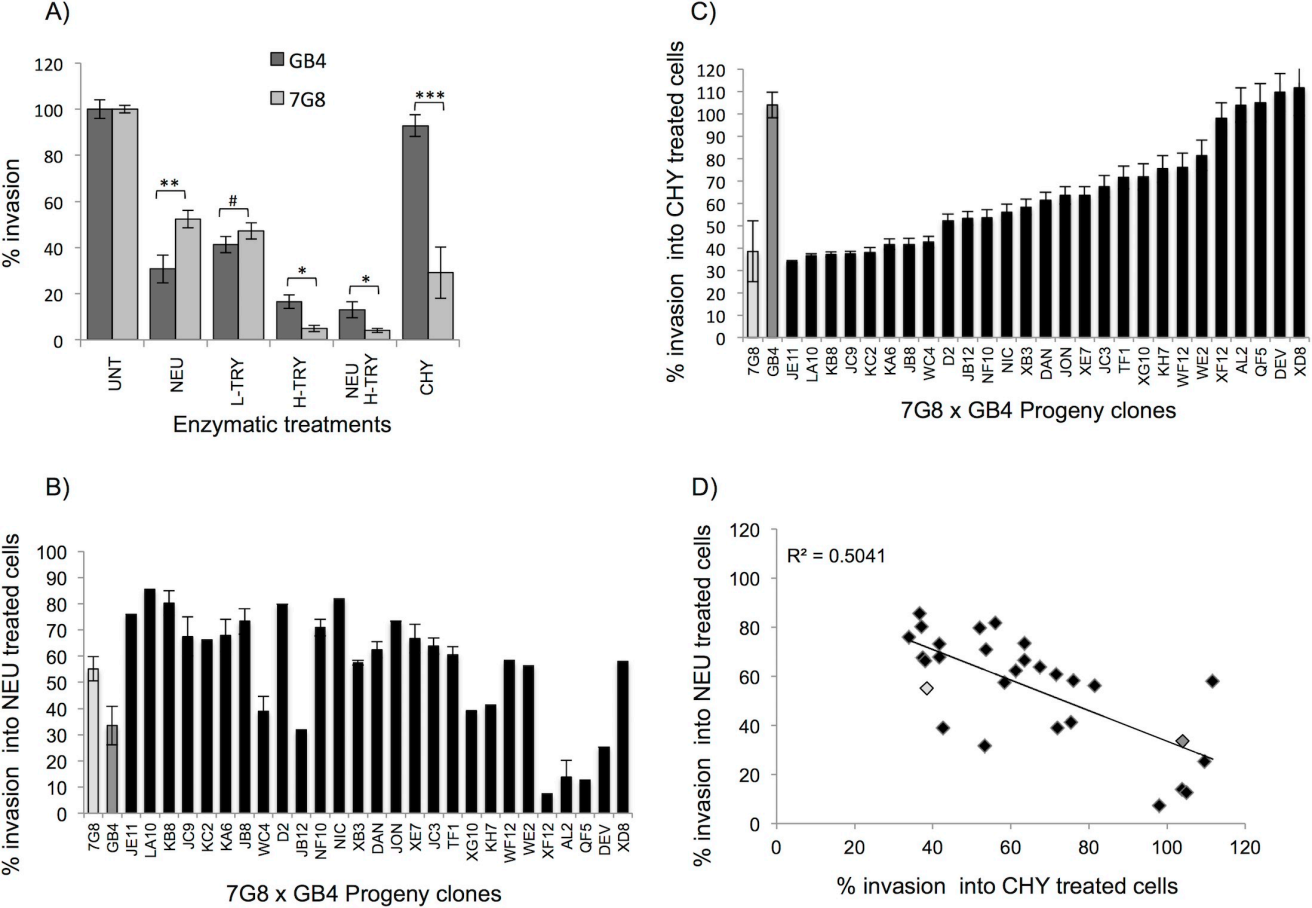
Erythrocyte invasion efficiency of progeny clones of the 7G8 x GB4 cross. (A) Invasion rates of the 7G8 (light grey bars) and GB4 (dark grey bars) parental strains into enzyme treated or untreated cells. # p-value = 0.25, *p-value≅ 0.012, ** p-value = 0.003, *** p-value = 0.00013. (B, C) Invasion profiles of 27 progeny clones (black bars) and parental strains (light grey: 7G8, dark grey: GB4) into NEU (B) and CHY treated (C) cells. Percentage values are relative to invasion into untreated cells. Results represent mean values from a minimum of 3 biological and 3 technical replicates. Error bars are standard error of the mean. (D) Correlation of mean invasion rates of progeny (black diamond) and parental (7G8: light gray, GB4: dark gray) into NEU and CHY treated erythrocytes.

The parental strains of the 7G8 (Brazil) x GB4 (Ghana) experimental genetic cross were phenotyped using a two-colour assay that sensitively measures parasite invasion into enzyme treated and untreated erythrocytes [30]. There was little difference in the ability of the two parental strains to invade erythrocytes treated with trypsin (TRY) at either high or low concentrations (Fig 1A), but there was a major difference in their ability to invade chymotrypsin (CHY)-treated erythrocytes. CHY treatment had no detectable effect on GB4 invasion, but reduced invasion of 7G8 parasites by >60% (p-value = 0.00013). By contrast, neuraminidase (NEU) treatment appeared to affect the GB4 parental line more than 7G8 (p-value = 0.003) (Fig 1A).

To establish whether these phenotypes are genetically inherited, the same assay was used to phenotype all 27 available independent recombinant progeny clones from the 7G8 x GB4 genetic cross. The invasion phenotypes of the progeny clones were variable, with many intermediate between the two parental lines (Figs 1B, 1C and S1), implying that these enzyme-sensitive invasion pathways are complex multigenic traits. However, ordering clones based on the effect of CHY treatment showed evidence of discrete groups, with the phenotypes of some clones being similar to 7G8 (JE11-WC4 in Fig 1C), others similar to GB4, while the remainder had intermediate phenotypes. This suggests that at least in the case of a CHY-responsive pathway, there may be one or two genes with strong effect sizes underlying this complex phenotype. Given the inverse link between CHY and NEU invasion observed in the parental clones, we ranked the clones in ascending order based on their CHY phenotypes, and assessed the sensitivity of invasion to NEU treatment (Fig 1B). The two invasion phenotypes showed a clear negative correlation across all clones (*R*^*2*^ = -0.504, Fig 1D). While this relationship was not absolute, of the five progeny clones least affected by CHY treatment, four were highly affected by NEU treatment (XF12-DEV in Fig 1B), indicating that the same gene(s) may underpin both phenotypes within this cross. Some of the other phenotypes were also correlated (S2 Fig), indicating that the same genes may underly multiple pathways in this cross.

The parents of another genetic cross, Dd2 (Laos) and HB3 (Honduras) are known to differ significantly in their susceptibility to NEU treatment [12], so we also tested the parents and progeny of this cross using the same assay. The effect of NEU of the progeny clones followed an almost continuous distribution between the parental phenotypes (S3 Fig) and subsequent QTL analysis identified no significant loci associated with either the NEU or CHY variable invasion phenotype (S4 Fig). This null result could be the combined effect of several genes each with small effect sizes, or epigenetic regulatory mechanisms that escape detection by the sequencing and analysis methods used here, and invasion phenotypes were not pursued further in this cross.

### Genetic mapping identifies a major locus on chromosome 13 that controls invasion pathways in the 7G8xGB4 cross

**Fig 2:**
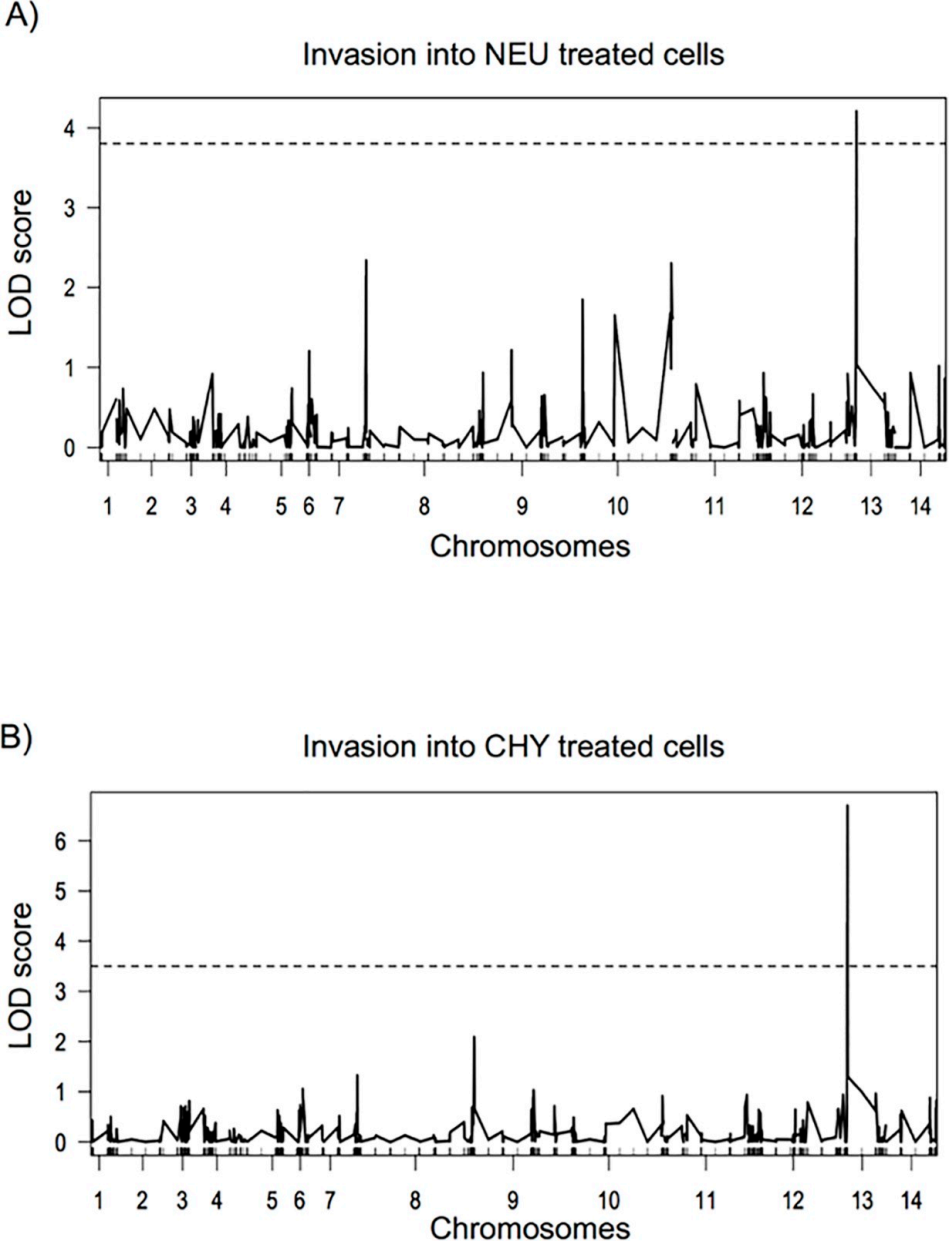
Genome-wide scan to detect quantitative trait loci (QTL) associated with erythrocyte invasion in the 7G8xGB4 cross. Logarithm of odds (LOD) score results for (A) NEU and (B) CHY invasion phenotypes, correlating with 5,433 SNPs across the genome generated by whole genome sequencing data. The dashed line represents the significant threshold (95%) based on 1000 permutations of the data.

Illumina sequencing data from the parents and progeny of three *P*. *falciparum* experimental genetic crosses have been previously used to call single nucleotide polymorphisms (SNPs), insertion/deletions (indels) and copy number variation (CNVs) with high accuracy [31]. We used QTL mapping with a curated set of SNPs from this dataset to search for loci associated with all four invasion phenotypes in the 7G8xGB4 cross. A genome-wide scan identified a locus on chromosome 13 with a highly significant LOD score for both NEU and CHY treatment phenotypes (NEU LOD = 4.2, CHY LOD = 6.7; Fig 2). The same locus was identified for both phenotypes, and variation within this locus explains the majority of phenotypic variation for both CHY (68.9% of observed variation) and NEU (51.2% of observed variation) treatment phenotypes. No other statistically significant loci were identified for CHY treatment. For NEU treatment, several minor peaks were detected on chromosomes 7, 9 and 10 but none passed the whole genome significance threshold, and only the minor peak on chromosome 10 (discussed below) contained genes previously associated with invasion (Fig 2). No significant loci were identified for the remaining enzyme treatment phenotypes. There was limited variation amongst clones after High-TRY treatment, which affected our ability to identify genetic signals. By contrast there was extensive variation after Low-TRY treatment, but no QTL signal was detected.

**Fig 3:**
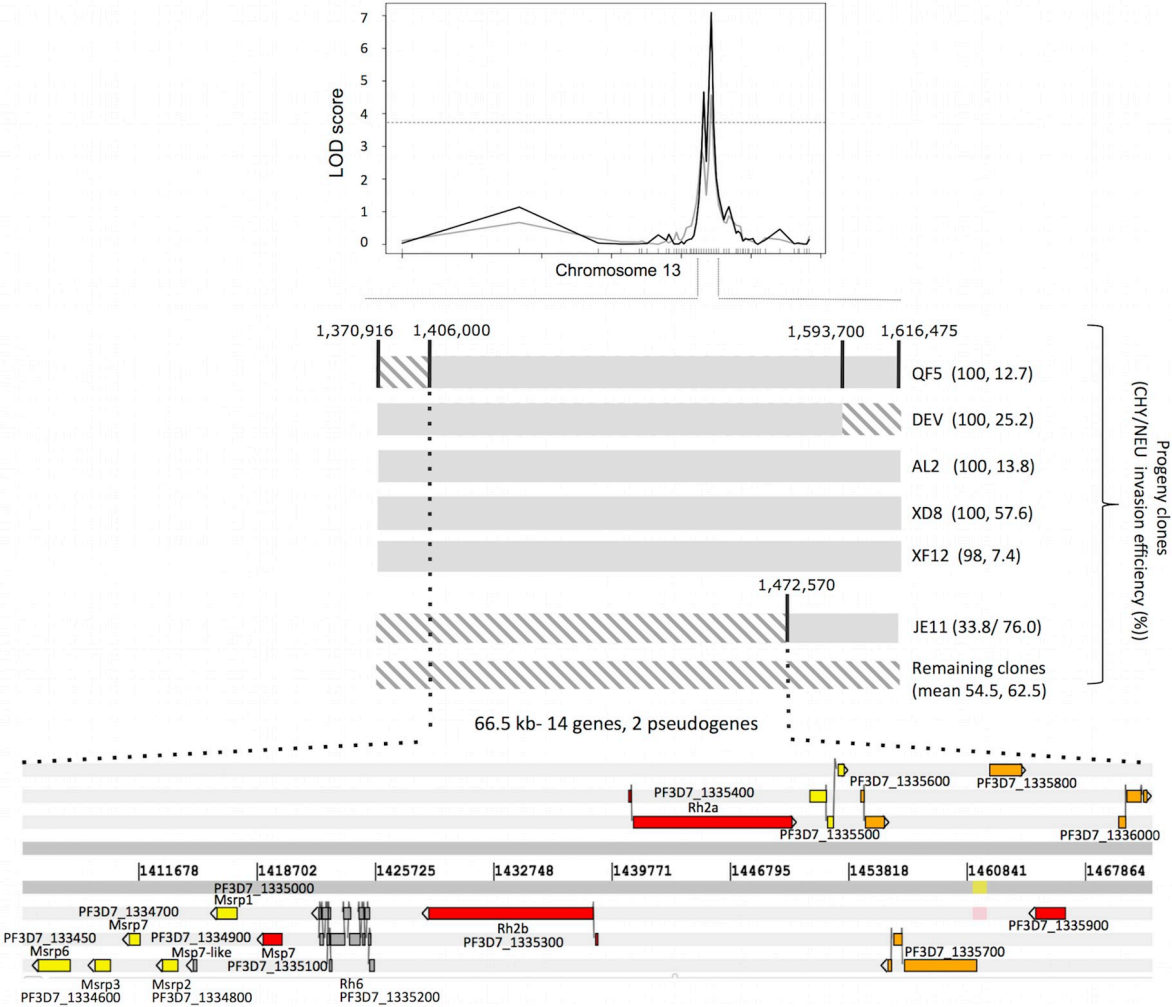
Fine mapping the invasion-associated QTL on chromosome 13. Map of the chromosome 13 region linked to CHY and NEU invasion phenotypes. By comparing the chromosomal segments inherited from each parental strain in the progeny clones (solid grey segments represent GB4 genome sequence, open diagonal grey segments represent 7G8 genome sequence) with their invasion phenotypes (phenotypes in brackets are the mean invasion rates into CHY, NEU treated erythrocytes, as shown in Fig 1), the region responsible for determining these two invasion phenotypes can be narrowed down to a 66.5kb fragment containing 14 genes.

To fine map the major locus on chromosome 13 underpinning the NEU and CHY treatment phenotypes we used Illumina sequence data to identify recombinant crossover breakpoints in the region (Fig 3) and compared SNPs and phenotype data between the different progeny clones to identify crossovers. This approach narrowed down the locus to a region of 66.57kb, located between 1.406 and 1.473 Mb on chromosome 13. Five progeny clones have the GB4 parental allele across this region, and all have identical CHY phenotypes to the parent GB4, with erythrocyte invasion completely unaffected by CHY treatment. Four of these same clones are also those most strongly affected by NEU treatment, as highlighted in Fig 1C. None of the remaining clones were completely unaffected by CHY treatment, and all carry the 7G8 allele at this locus. These results show that this 66.57kb segment contains the primary determinants of both NEU and CHY invasion phenotypes in this genetic cross.

To search for loci that could explain the remaining unassigned fraction of variation for the NEU and CHY phenotypes, a secondary genome-wide scan was conducted, controlling for the main effects on chromosome 13 identified in the primary scan. No other genomic regions reached statistical significance for the CHY treatment phenotype, suggesting that the chromosome 13 locus contains the primary gene(s) for this phenotype in these genetic backgrounds. For the NEU treatment phenotype, a region on chromosome 10 identified as a minor locus previously became statistically significant (LOD = 4.1, threshold = 3.6, 29.3% of remaining observed variation). This locus was mapped to a region between 1377067bp and 1569779bp, containing 57 genes, several very polymorphic, including the Merozoite Protein 3 (*PfMSP3*) gene family (S5 Fig), two of which (*PF3D7_1035700* and *PF3D7_1036300*) have been shown to bind erythrocytes [32, 33]. However, given the number of genes within this secondary locus, and its relatively low effect size on only one phenotype, we focused our subsequent analysis on the major locus on chromosome 13 affecting both NEU and CHY phenotypes.

### Long read sequencing identifies a deletion between the *Plasmodium falciparum* Reticulocyte Binding Protein Homologue 2a and 2b genes

The locus on chromosome 13 contains fourteen annotated genes and two pseudogenes (Fig 3). Seven of these genes are expressed during late stages of the intra-erythrocyte cycle (>35 hours), including several previously implicated in erythrocyte invasion, most notably *P*. *falciparum* Merozoite Surface Protein 7 (*PfMSP7* and a cluster of *PfMSP7*-related genes, and two genes encoding invasion ligands from the *P*. *falciparum* Reticulocyte Binding Protein Homologue family (*PfRh2a* and *PfRh2b*). Illumina sequencing data identified only 5 SNPs that differed in *PfMSP7* between the two parental clones, and none in any of the *PfMSP7* homologues.

Calling variation in *PfRh2a* and *PfRh2b* genes is more complex, as the two genes are positioned head-to-head and are identical over >8kb of their sequence, differing only at their 3’ ends, which encodes the carboxy-terminal 400–500 amino acids of the expressed proteins [34]. As a result, it is impossible to assign short sequence reads unequivocally to one gene or the other through the majority of their length. To identify polymorphisms specific to each gene, we sequenced each gene in the GB4 and 7G8 parental strains using Sanger/capillary technology. We also used PacBio whole genome sequencing data for these two strains to obtain long fragment reads that cover both genes [35]. These approaches identified several SNPs and two deletions in the GB4 strain. One of the deletions, spanning 156bp, was present in a repeat region in the *PfRh2b* gene close to the 3’ end, which marks the boundary between the region shared by *PfRh2a/2b* and the region unique to *PfRh2b*. Indels in this region have been reported previously and do not affect the reading frame of the encoded protein [36, 37]. A novel deletion (681bp) was identified in the non-coding region between the *PfRh2a* and *PfRh2b* genes. This deletion could only be identified in PacBio data emphasizing the difficulty of calling variants in the extremely AT-biased non-coding regions of the *P*. *falciparum* genome. Using the PacBio data as a reference for Illumina mapping, we confirmed that this deletion is present in all five clones that have the same CHY phenotype as GB4 and which inherited the GB4 chromosomal segment, while it was absent from all clones that inherited the 7G8 chromosomal segment.

### Presence or absence of an intergenic deletion correlates with differential expression of *PfRh2a* and *PfRh2b* and differential invasion phenotypes

**Fig 4:**
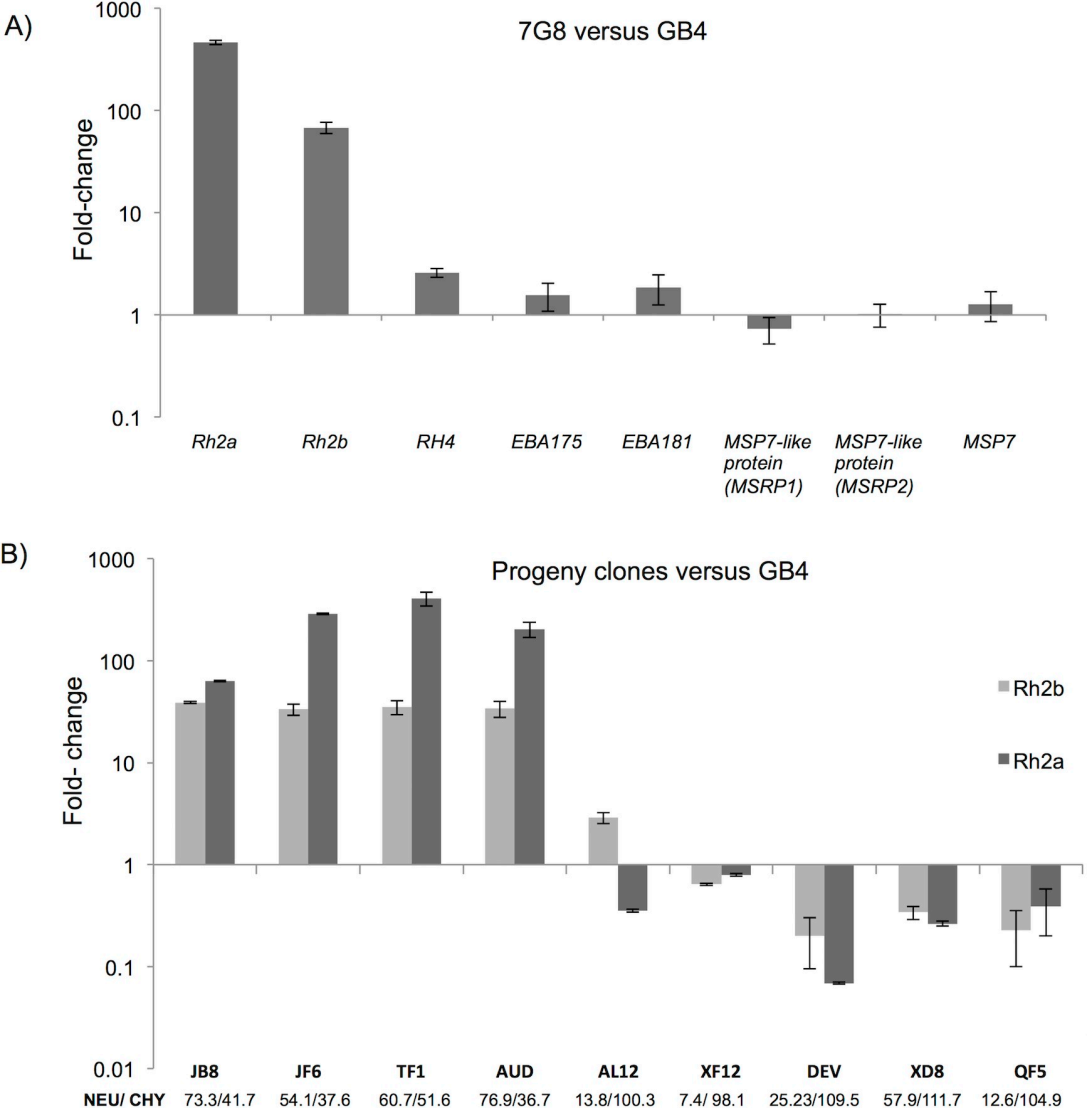
Relative transcript levels of genes mapping at the QTL on chromosome 13. (A) qRT-PCR data from the two parental strains, 7G8 and GB4, measuring expression of five genes expressed during schizont stage and present within the QTL associated with CHY and NEU invasion phenotypes (*PfRh2a*, *PfRh2b* and 3 genes from the *PfMSP7* family). Two invasion related genes (*PfEBA175*, *PfEBA181*) were tested as controls. Expression of all genes was normalized to *PfAMA1*. (B) Expression of *PfRH2a* and *PfRh2b* in nine progeny clones, four in which invasion is affected by CHY treatment (JB8, JF6, JE1, AUD) and five where CHY has no effect on invasion (AL2, XF12, DEV, XD8, QF5). Expression was measured using qRT-PCR as above, and is displayed relative to expression in the GB4 parental line. Mean invasion rates for each progeny into NEU/CHY treated erythrocytes are listed under the clone name.

This deletion in the *PfRh2a/2b* promoter region has not been previously reported and due to its position, we hypothesized that it might affect transcription of one or both genes. Quantitative RT-PCR was used to quantitate expression of *PfRh2a*, *PfRh2b*, members of the neighboring *PfMSP7* family as well as control invasion genes. Comparing gene expression between the two parental lines, 7G8 and GB4, revealed a >80-fold decrease in the expression of *PfRh2a* and *PfRh2b* in GB4 relative to 7G8 (Fig 4A). At an absolute level, there was almost no expression of *PfRh2a* in GB4 parasites, and expression of *PfRh2b* was extremely low. By contrast, there was no significant difference in expression of *PfMSP7* or two other neighboring *PfMSP7*-like genes between the two parental lines (Fig 4A). To test whether the differences represented a general shift in the expression of invasion ligands, we compared gene expression between the lines for three other major invasion ligands previously associated with alternative invasion pathways, *PfEBA175*, *PfEBA181* and *PfRh4*. All showed only minor variation, with expression increased 1.6- (*PfEBA175)*, 1.8- (*PfEBA181)* and 2.5- (*PfRh4)* fold in 7G8 relative to GB4. Extending the analysis to progeny clones from the 7G8xGB4 genetic cross showed that all five progeny that had inherited the *PfRh2a/2b* intergenic deletion found in GB4 also had very similar *PfRh2a* and *PfRh2b* expression levels to GB4 (AL12-QE5, Fig 4B), with transcription of both genes almost absent. By contrast, four other clones that lack the intergenic deletion, like 7G8, all had *PfRh2a* and *PfRh2b* expression levels comparable to the 7G8 parent (JB8-AUD, Fig 4B), much higher than GB4. There is therefore a clear and strong association between a novel indel in the *PfRh2a* and *PfRh2b* intergenic region, specific and extreme down-regulation of expression of these two genes, and use of a CHY-resistant and NEU-sensitive invasion pathway.

In order to confirm the role of *PfRh2a* and *PfRh2b* expression in the invasion phenotype differences between 7G8 and GB4, we used a CRISPR/Cas9 strategy to modify these genes in the 7G8 parent parasite line. Because the deletion in the GB4 parent line affects expression of both genes, we took advantage of the identity between *PfRh2a* and *PfRh2b* to target a resistance cassette to the coding region common to both genes, in a dual disruption strategy (S6 Fig). After selection, drug resistant parasites were cloned and genotyped by PCR using a forward primer placed in the resistance cassette and reverse primers specific for the unique regions of either *PfRh2a* or *PfRh2b*. Genotyping established that all clones had integrated the resistance cassette in both genes (S7 Fig). This integration was confirmed by whole genome sequencing using Illumina technology; coverage plots showed an absence of reads aligning to the central region of each gene, corresponding to the locations targeted for deletion (S8 Fig).

**Fig 5:**
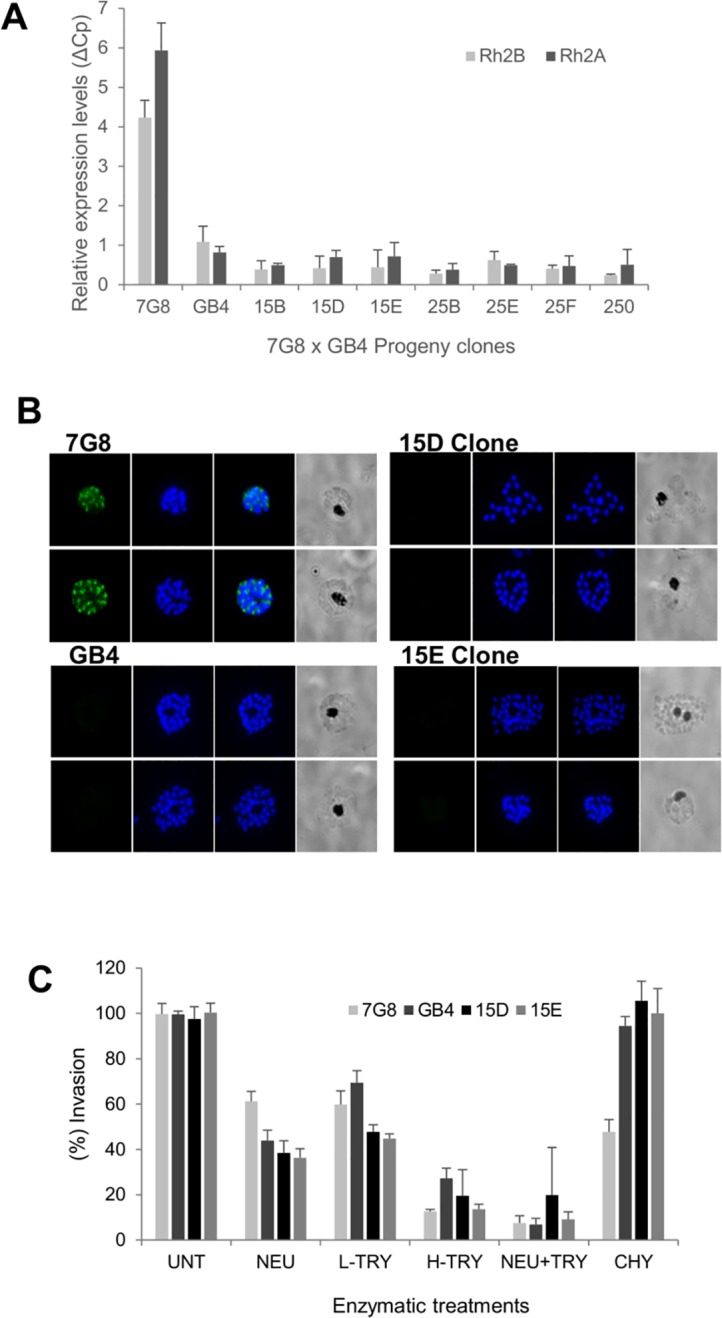
Gene editing and phenotyping of *P*. *falciparum* 7G8 strain to eliminate *PfRh2a* and *PfRh2b* expression. *PfRh2b and PfRh2a* were targeted in 7G8 using CRISPR/Cas9 technology and several clones were obtained from the resulting transfectant population. A) Expression of *PfRh2b and PfRh2a* genes for the parental lines 7G8 and GB4 and the edited 7G8 clones. B) Immunofluorescence analysis of the parental lines 7G8 and GB4 and two of the edited 7G8 clones using a specific anti-Rh2b antibody (green), nuclei are labelled with DAPI (blue). C) Invasion phenotypes of the edited 7G8 clones and the parental lines into erythrocytes untreated (UNT) or treated with neuraminidase (NEU), low trypsin (L-TRY), high trypsin (H-TRY), neuraminidase + trypsin (NEU+TRY) and chymotrypsin (CHY).

Expression of both *PfRh2a* and *PfRh2b* in the edited clones was greatly decreased compared to the parental line 7G8 and matched levels detected in GB4 (Fig 5A), suggesting the dual disruption strategy recapitulated the expression effect of the intergenic deletion in the GB4 parent. Expression of PfRh2b protein was investigated in the parent strains and the edited clones by immunofluorescence, which showed clear PfRh2b labeling in 7G8, but with no detectable protein in GB4 or the edited clones (Fig 5B). To establish whether disruption of these genes impacted the pattern of erythrocyte invasion pathway usage, the two disrupted clones were phenotyped using the two-colour invasion assay. Both clones had phenotypes similar to the GB4 strain, being significantly more CHY-resistant and NEU-sensitive than their parental 7G8 line (Fig 5C). Deletion of *PfRh2a* and *PfRh2b* in 7G8 therefore phenocopies the invasion preferences of GB4, and confirms that these genes are responsible for the major variation in alternative invasion pathway usage between these two lines. It should be noted that the strategy used here, by recapitulating the GB4 parent and decreasing expression of both *PfRh2a* and *PfRh2b*, cannot distinguish between individual effects of these two genes.

### Indels in the *PfRh2b/ PfRh2a* locus are frequently found in clinical *P*. *falciparum* isolates

**Fig 6:**
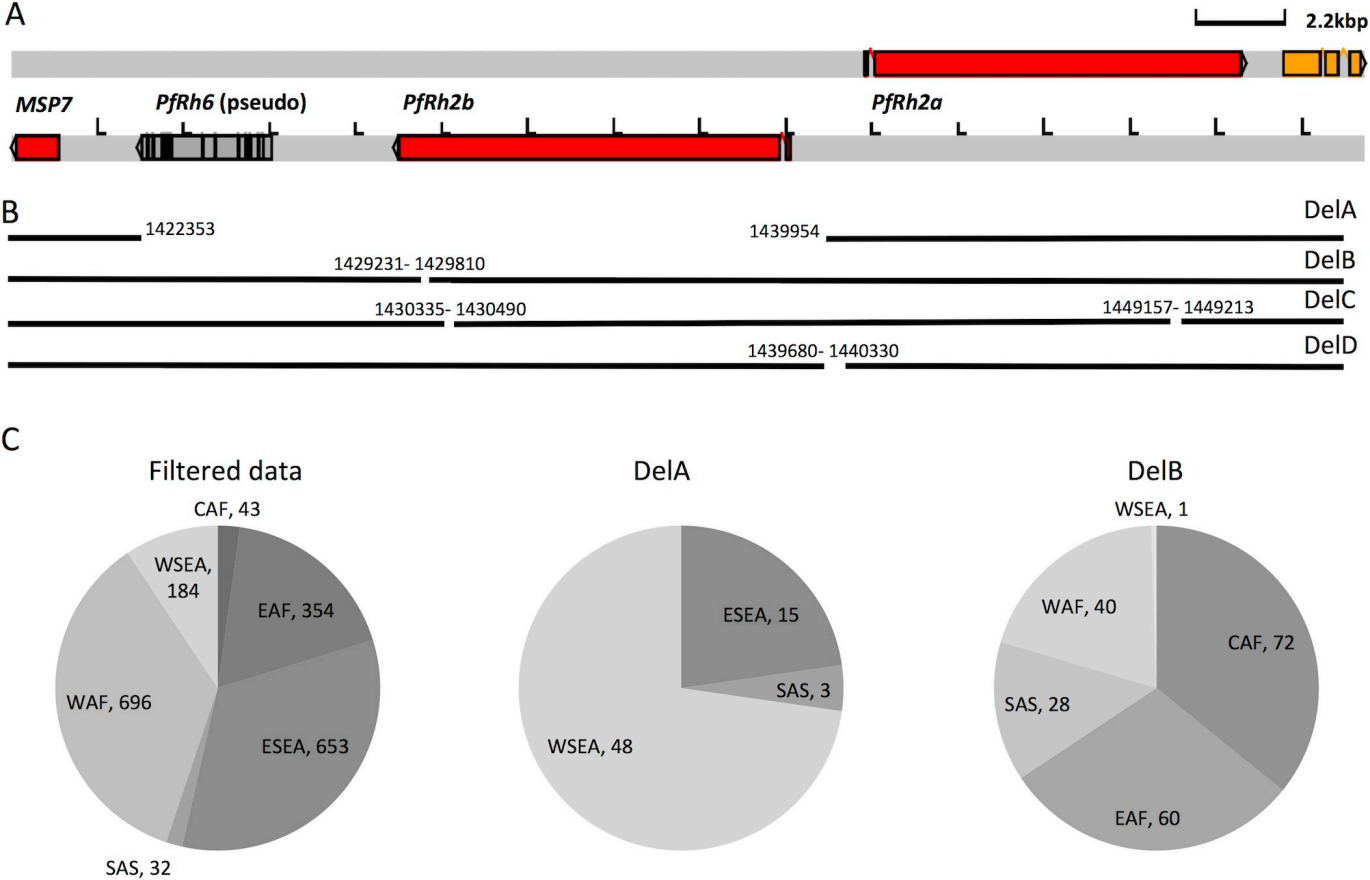
Large insertions and deletions at the *PfRh2a/PfRh2b* locus are common and globally distributed. (A) Map of genomic region around *PfRh2a* and *PfRh2b*. (B) Graphical delineation of the deletions detected using Pacbio sequencing data from 15 strains sequenced as part of the Pf3k project. A deletion of the entire *PfRH2b* gene along with the neighbouring pseudogene *PfRh6* (Del A), deletion within the unique region of *PfRh2b* (Del B) or repeat regions of *PfRh2b and PfRh2a* (Del C) which all maintain the translation frame, and an intergenic deletion (Del D). (C) Global prevalence of PfRh2b indels across field isolates sequenced as part of the MalariaGEN project; (left) the total number of clinical samples from each geographical region for which *PfRh2b* indels could be counted (WAF: West Africa; EAF: East Africa; CAF: Central Africa; SAS: South Asia; ESEA: East Southeast Asia; WSEA: West Southeast Asia), (middle) frequency of clinical isolates with DelA and (right) frequency of clinical isolates with DelB.

These data suggest that a previously undiscovered mechanism, a deletion within the *PfRh2a/2b* intergenic region, affects expression of these genes and can radically influence invasion phenotypes. Establishing whether such deletions are also present in clinical isolates is complicated by the fact that, as noted above, the large extent of identical sequence shared by both genes affects read mapping and hence indel calling. The presence of multiple strains within clinical infections, such as occurs commonly in high transmission regions, would also significantly impact mapping. We therefore first analysed 15 high quality genomes that had been generated from long-read PacBio data, which includes both laboratory and field isolates [35]. One isolate (KH01) had a deletion of the entire *PfRh2b* gene as well as a neighboring pseudogene *PfRh6* (Fig 6 DelA), a significant change that has been previously identified in clinical isolates [36]. A second previously identified deletion was also confirmed in three PacBio sequenced samples (KE01, GA01, GN01); a 585bp deletion in the unique 3’ end of *PfRh2b* that maintains the PfRh2b reading frame [37] (Fig 6 Del B). Deletions in the repeat regions between the shared and unique regions of Pf*Rh2a* and *PfRh2b* (Fig 6 Del C) were also present in multiple isolates. Finally, a larger intergenic deletion (676bp), partially overlapping the region with the deletion present in GB4, was identified in the IT strain (Brazil) (Fig 6 Del D). Searching publicly available Illumina data from the Pf3k project revealed that more than 10% of clinical isolates had one of these deletions, making indels at this locus very common. Markedly different distributions were observed, with the deletion of the whole *PfRh2b* gene (Del A) present exclusively in Asia, and the deletion in the unique region of *PfRh2b* (Del B) found primarily in Africa. Accurate estimation of intergenic deletions such as Del D with Illumina data is difficult due to the repetitive nature of the sequence and the high AT content noted above, so frequency of Del D or other intergenic deletions could not be estimated.

## Discussion

Differential usage of alternative invasion pathways is one of the most widely described variable phenotypes in clinical *P*. *falciparum* isolates, alongside drug resistance and cytoadherence. Previous attempts to identify the genes responsible for specifying alternate invasion have almost exclusively taken a reverse genetics approach, interrogating candidate genes one by one. These studies have been highly informative, but given the likely multigenic nature of the phenotypes, will always lead to only partial conclusions about the relative importance of different genes. We have applied forward genetics to this problem for the first time, using the parents and progeny of a *P*. *falciparum* experimental genetic cross. Combining sensitive phenotyping of invasion into enzyme treated erythrocytes with Illumina sequencing data revealed a single locus responsible for 69% of variation in invasion into chymotrypsin (CHY) treated erythrocytes, and 51% of variation in invasion into neuraminidase (NEU) treated erythrocytes. This locus contained 15 genes, including the *PfRH2a* and the *PfRh2b* genes. The phenotypes correlated with the presence of a deletion in the intergenic region between the two genes, which lie head to head on chromosome 13, and with a concomitant severe down-regulation of expression of both genes. The primary role of these genes was confirmed by CRISPR-Cas9 genome editing, where disruption of the *PfRh2a* and *PfRh2b* genes converted the 7G8 strain to a GB4-like invasion phenotype.

While forward genetics is by definition not limited to studying only previously identified candidate genes, it is striking that the unbiased forward genetics approach identified two candidate genes, *PfRh2a* and *PfRh2b*. However, while *PfRh2a* and *PfRh2b* have been associated with alternative invasion in the past, the central nature of their role in this case would not necessarily have been predicted. The strongest candidate might have been predicted to be the sialic acid dependent ligand, EBA-175, which binds to its receptor Glycophorin A in a NEU sensitive and CHY resistant manner [5] and when deleted leads to a switch to NEU-resistant invasion pathways [9], but we found no evidence for an association at this locus.

Our data does however fit with previous experimental studies that have focused on the *PfRh2a/2b* locus. Initially associated with invasion nearly two decades ago [34, 38], both PfRh2a and PfRh2b are extensively proteolytically processed, and bind to erythrocytes in a chymotrypsin sensitive and neuraminidase resistant manner [39, 40]. Antibodies raised against both proteins can inhibit erythrocyte invasion, and it has been extensively studied as a potential vaccine candidate [39, 41]. In this study, low *PfRh2a/2b* expression was associated with an increase in invasion into CHY-treated erythrocytes and a decrease in invasion into NEU-treated erythrocytes. Deleting the *PfRh2b* gene in both the sialic acid-dependent 3D7 strain and the sialic acid-independent W2mef strain resulted in very similar phenotypes, increasing invasion into CHY-treated erythrocytes and decreasing invasion into NEU-treated erythrocytes [8, 42]. Deletion of *PfRh2b* alone is also associated with changes in CHY and NEU invasion phenotypes, and domain swap experiments subsequently revealed that it is specifically the transmembrane and cytoplasmic domains of PfRh2b that are functionally important in specifying variable invasion pathways [43]. Therefore, while the indel identified in our QTL analysis is associated with decreased transcription of both *PfRh2a* and *PfRh2b*, it is likely that it is specifically the decrease in *PfRh2b* expression that leads to the change in invasion phenotypes in this genetic cross.

These results also concur with previous studies of variation in *PfRh2a* and *PfRh2b* in clinical isolates. A naturally occurring 582bp deletion in the unique region of PfRh2b, first identified in Senegalese *P*. *falciparum* strains and then found to be common in multiple locations around the world [37], has been associated with decreased invasion into NEU-treated erythrocytes, although only among parasites isolated from blood group O donors [20]. In addition, single nucleotide variants and other smaller indels within *PfRh2b* have been associated with NEU sensitive invasion in Brazilian isolates [19, 44] and decreased *PfRh2b* expression levels have been associated with an increase in CHY resistant invasion in Tanzanian isolates [22]. There is therefore strong evidence that variation at *PfRh2b* in clinical isolates is widespread and likely to play a primary role in determining the CHY and NEU alternative invasion pathways.

*P*. *falciparum* genetic crosses offer a robust tool to identify loci that have a large effect size on a given phenotypic trait. However, the number of progeny for each cross is relatively small, which reduces the statistical power to identify loci with small effects. Controlling for variation at the major locus on chromosome 13 identified a second locus on chromosome 10 that was specifically in linkage with the NEU treatment phenotype, but not the CHY treatment phenotype. This locus contains most notably the *PfMSP3* gene family [45], two members of which, MSP3DBL1 (MSP3.4) and MSP3DBL2 (MSP3.8) have been shown to bind erythrocytes [32, 33] as well as IgM [46]. These genes are amongst the most polymorphic in the *P*. *falciparum* genome and are under extremely strong balancing selection, which may be due to host acquired immunity or interaction with variable erythrocytes receptors [47]. While these, and other genes within the *PfMSP3* family, have been widely associated with invasion, they have not been previously implicated in specifying alternative invasion pathways, emphasizing the utility of unbiased forward genetics for exploring such multigenic traits. Confirmation of which genes within this large locus contribute to invasion into NEU treated erythrocytes will require further work, but members of the *PfMSP3* gene family will be the obvious place to start.

The suggestion that alternative pathways are generally multigenic is emphasized by the fact that for several phenotypes, progeny clones were more affected by enzyme treatment than either parent (Fig 1C, S1 Fig). While this could be because there are multiple genes each of which has a minor effect, it might also indicate that epigenetic effects may be involved and would not be detected using our sequence-based approach. At least some invasion-associated genes are known to be clonally variant [48], with repression of invasion genes including *PfRh4* associated with H3K9me3-based heterochromatin [49], and activation associated with H3K9ac [50]. Knockdown of a specific bromodomain protein predicted to bind H3K9ac, PfBDP1, results in up-regulation of a wide range of invasion-associated genes, including *PfRhs* and *PfEBAs* [51]. Epigenetics therefore clearly play a strong role in regulating invasion associated-genes, and as the progeny of a genetic cross have gone through both mosquito and liver stages after the parental lines were crossed, there has been ample opportunity for resetting of the epigenetic code. Given this, the fact that the *PfRh2b/ PfRh2a* effect reached genome-wide significance in the 7G8xGB4 cross is in some ways surprising, and indicative of its importance. Alternative approaches, such as characterizing the transcriptome, histone modifications and epigentic landscape, may be required to uncover other genes underpinning alternative invasion in future studies.

While large-scale systematic studies will be needed to completely disentangle the relative contribution of all genes involved, both reverse and forward genetic studies, and lab and field studies, are clearly converging on *PfRh2b* as a primary ligand controlling alternative invasion phenotypes. Variations within the *PfRh2b* gene exist globally, some strongly differentiated in frequency between geographic regions. The receptor for PfRh2b is not known, and it is therefore not clear what selection pressure is driving differentiation at the *PfRh2b* locus, but it is presumably linked to variation in the expression or sequence of an erythrocyte surface protein. Establishing the specific host-parasite interaction involved will be the critical next step in order to establish why variable erythrocyte invasion is such a common, and globally distributed, phenotype among *P*. *falciparum* strains, and to inform the design of strain-transcending vaccines.

## Materials and methods

### Ethics statement

Erythrocytes for culture of *Plasmodium falciparum* parasites were sourced from NHS Blood and Transplant, Cambridge, UK. All samples were anonymized. Use of erythrocytes from human donors for *P*. *falciparum* culture was approved by the NHS Cambridgeshire 4 Research Ethics Committee (REC reference 15/EE/0253) and the Wellcome Trust Sanger Institute Human Materials and Data Management Committee.

### *In vitro* culture of *P*. *falciparum*

*P*. *falciparum* clones from the 7G8 x GB4 (n = 27) and HB3 x Dd2 (n = 35) crosses were kindly provided by Dr. Karen Hayton, Professor Thomas Wellems and Dr Mike Ferdig. Parasites were cultured in complete medium containing 10% human sera, at 5% hematocrit in O+ erythrocytes. Cultures were maintained at 37°C under an atmosphere of 1% O2, 3% CO2, and 96% N2. Before the performance of the invasion assay, parasite cultures were synchronized with 5% D-sorbitol (Sigma-Aldrich, Dorset, UK). DNA extraction for sequencing was undertaken using the QIAamp DNA Blood Midi Kit (Qiagen) or the Genomic tip (Qiagen) to obtain high-molecular-weight DNA.

### Invasion assays

Erythrocyte invasion assays were performed as described previously [30]. Briefly, uninfected erythrocytes were treated with proteolytic or glycolytic enzymes to remove a subset of cell-surface receptors: neuraminidase (NEU) (final concentration of 20 mU/mL), trypsin (TRY) (50 μg/mL for low trypsin or 1 mg/mL for high trypsin), chymotrypsin (CHY) (1 mg/mL) and a combination of NEU and TRY. Treated erythrocytes were labeled with the intracellular dye DDAO-SE (Invitrogen), washed and incubated for 48 hours with an equal volume of *P*. *falciparum* ring stage parasites (c. 2% parasitemia). After co-incubation, parasites were labeled with the fluorescent DNA-intercalating dye Hoechst 33342 (Invitrogen), and parasites that had invaded labeled cells quantified by two color flow cytometry (BD LSRII- BD Biosciences). Stained samples were examined with a 355 nm UV laser (20 mW) and a 633 nm red laser (17 mW). Hoechst 33342 was excited using the UV laser and detected with a 450/50 filter and the DDAO-SE was excited using the red laser and detected with a 660/20 filter. A total of 100,000 events were collected for each sample. The data collected was analyses with FlowJo. Three technical replicates were performed for each enzyme/strain combination (i.e. three wells of the same combination performed in a given assay), and a minimum of 2 biological replicates were performed (i.e. each strain was assayed at least twice, on two different days). Data is presented as the mean ± the standard error of the mean. Invasion efficiency is represented as the percentage of invasion into enzyme treated labeled cells relative to the percent invasion into untreated labeled cells.

### Genome sequencing

All parent and progeny samples were sequenced on the Illumina Genome Analyzer platform (www.illumina.com) at the Wellcome Sanger Institute. QTL analysis was performed using a subset of quality-controlled SNPs (86,158 SNPs) that were selected from an initial set of potential SNPs by applying a series of quality filters, as described [52], as well as a subsequent set of SNPs called specifically on these experimental genetic crosses [31]. The *PfRh2a* and *PfRh2b* genes were sequenced by Sanger / capillary technology using specific primers that amplify each gene independently [53]. Fifteen *P*. *falciparum* strains, including the 7G8 and GB4 parental strains were also sequenced using the PacBio sequencing system as part of the Pf3k Project (https://www.malariagen.net/projects/pf3k). Genome sequence data are available in ftp://ftp.sanger.ac.uk/pub/pathogens/Plasmodium/falciparum/PF3K/PilotReferenceGenomes/DraftAnnotation/ and is summarized in [35]. Illumina data for 2,500 *P*. *falciparum isolates*, part of the Pf3k project, was screened to identify deletions in the *Pfrh2b* and *PfRH2a* genes.

### QTL analysis

Genome wide linkage scans were performed using 5,433 SNPs for the 27 clones of the 7G8 x GB4 cross and 4,407 SNPs for 30 clones of the HB3 x Dd2 cross. Five clones within the latter cross were removed due to low sequence coverage. The SNP sets were all polymorphic between the parental clones, and there were at least two minor alleles in progeny clones. QTL analysis was performed using the *R/QTL* software (www.rqtl.org). An average recombination of 13.5 kb/cM has been described for the crosses used here [31], giving an estimate of at least 3 markers per uniquely inherited genome segment (excluding telomeres, centromeres and sub-telomeres). Thresholds for statistical significance across the genome scans were determined using permutation analysis. To identify any additional loci, secondary scans were performed and the models included loci identified in the first QTL.

### Gene expression

Synchronized cultures enriched in schizonts were used to obtain RNA from the samples. The Ambion Ribopure blood kit and Isolate II RNA mini kit (Bioline, UK) was used to perform RNA extraction, followed by DNAse treatment and cDNA synthesis (Ambion). Gene expression for *PfRh2b*, *PfRh2a*, *PfAMA1*, *PfEBA175*, *PfEBA181* and *PfRh4* was measured using the conditions and primers/probes listed in [19] using qPCR Roche equipment and solutions. For *PfMSP7*, *PfMSRP1* and *PfMSRP2* primers and probes (labeled with 6-FAM) were: PfMSP7F: 5’- tgtcgattctcctccttg-3’, PfMSP7R: 5’- gcacaaagtgaaacagatac-3’, PfMSP7P: 5’- tcttgtccttgtgttgatatctcttgt-3’, PfMSRP1F: 5’- tcctcttggttgtgattc-3’, PfMSRP1R: 5’- gtcccgatgtatcatcaa-3’, PfMSRP1P: 5’- atgccagaatcaccaagaccaga -3’, PfMSRP2F: 5’- gtggtgtacttaaatttgatg-3’, PfMSRP2R: 5’- gggaatcagaagataatacaa-3’, PfMSRP2P: 5’- ccaaagtccaaggtgctcaagtt-3’. Relative differences in gene expression were calculated using the ΔΔCq method, with expression of the *PfAMA1* gene acting as a control to confirm that parasites were at a similar stage of development. The GB4 parental strain was used as the reference strain.

### Immunofluorescence assay (IFA)

Air-dried thin films of late-stage schizonts were fixed in 4% formaldehyde. Fixed parasites were permeabilised in 1% Triton X100/PBS and blocked in 3%BSA/ 10% goat serum/PBS. Parasites were probed with polyclonal rabbit anti-Rh2b (4D1, [34]) primary antibody at 1:200 overnight at 4°C. After three washes, the parasites were incubated with a fluorescent secondary antibody (Goat anti-Rabbit IgG (H+L) Highly Cross-Adsorbed Secondary Antibody, Alexa Fluor 488 #A11034) diluted 1:500 for 1 hr at room temperature. The samples were washed an additional three times and mounted in Prolong Gold (Molecular Probes) with DAPI. Antibody probing and wash steps used 3% BSA/PBS buffer. Images were captured on a Leica DMi8 fluorescent microscope and processed using Leica LAS X software and Photoshop.

### Long read assemblies and indel frequency calculation

The genome sequences (PacBio data) for GB4, 7G8 and the 13 other strains were mapped to the reference *P*. *falciparum* genome (3D7 version 3.0) using *bwa*-*mem* [54]. The alignments were visualized in *BAMview* [55] to identify indel variants. For field isolates, mapped Illumina reads from the Pf3k samples (n = 2,500) were counted within and surrounding candidate regions (e.g. *PfRH2a* and *PfRH2b* genes) using *Samtools* software [56]. The coverage counts were normalized to 100-fold for presentation. Deletions were scored by generating ratios of coverage between regions within and flanking the potential deletion. Score cut-offs were chosen based on empirical distributions, with calibration using known deletions, and accounting for any mixed infections.

### Genome editing

7G8 parasites were transfected by erythrocyte preloading [57]. Plasmids were obtained from PlasmoGEM: pCC1, containing the selection cassette, a barcode and homology arms for the common *PfRh2a/PfRh2b* region; pDC2 with two gRNAs targeting this region of the two genes and Cas9 (S6 Fig). 50μL of each plasmid were used to electroporate 300μL of packed RBCs at 0.31kV and 950μF with a Gene Pulser (BioRad, Watford UK). The transfected RBCs were mixed with a parasite culture of 5–6% parasitaemia containing approximately 1.5% schizonts. Targeted parasites were selected with 2.5nM WR99210 48 hours after transfection for 6 days and the culture was followed until the appearance of parasites. To ensure editing and eliminate plasmid episomes, the culture was treated with 2.5nM WR99210 together with 40μM 5FC for a week, after which the parasites were cloned by serial dilution. The clones obtained were confirmed by genotyping with the following primers and sequencing: CAM5 F 5’-ccaatagataaaatttgtagag-3’, AR1 R 5’-aggtttaatatcgacgagtc-3’, AR2R 5’-gaacatcatcattcggttc-3’, BR1 R5’-cgctttctgtaatttcactg-3’, BR2R 5’-ctagcatcacgttggtc-3’.

## Supporting information

S1 Fig Erythrocyte invasion efficiency of 7G8xGB4 cross progeny clones into trypsin treated cells. Invasion profiles of 27 progeny clones (black bars) and parental strains (light grey: 7G8, dark grey: GB4) into erythrocytes treated with (A) low trypsin (50 μg/mL) or (B) high trypsin (1 mg/mL). Percentage values are relative to invasion into untreated cells. Results represent mean values from a minimum of 3 biological and 3 technical replicates.
(JPG)

S2 Fig Correlations between invasion phenotypes. Correlation of mean invasion rates of progeny (black diamond) and parental (7G8: light gray, GB4: dark gray) into enzyme treated erythrocytes. All phenotypes were compared in a pair-wise manner: NEU/L-TRY (A), CHY/L-TRY (B), NEU-H-TRY (C), CHY-H-TRY (D) and L-TRY-H-TRY (E).
(JPG)

S3 Fig Erythrocyte invasion efficiency of progeny clones of the HB3 x Dd2 cross. (A) Invasion rates of the Dd2 (light grey bars) and HB3 (dark grey bars) parental strains into enzymatic treated and untreated cells. *p-value> 0.06, ** p-value< 0.00001. (B) Invasion profiles of 35 progeny clones (black bars) and parental strains into NEU (B) and Low(L)-TRY treated (C) cells. Percentage values are relative to invasion into untreated cells. Results represent a minimum of 2 biological and 3 technical replicates. Error bars are standard error of the mean.
(JPG)

S4 Fig Genome-wide scan to detect quantitative trait loci (QTL) associated with erythrocyte invasion in the HB3xDd2 cross. Logarithm of odds (LOD) score results for (A) NEU and (B) CHY invasion phenotypes, correlating with 5,433 SNPs across the genome generated by whole genome sequencing data. The dashed line represents the significant threshold (95%) based on 1000 permutations of the data. No loci reached genome wide significance.
(JPEG)

S5 Fig Genome-wide scan to detect quantitative trait loci (QTL) associated with NEU sensitive erythrocyte invasion, controlling for the major locus on chromosome 13. Logarithm of odds (LOD) score results for the invasion phenotype into NEU-treated (A) and CHY-treated (B) cells correlated with 5,433 SNPs across the genome generated by whole genome sequencing data, after variation at the major locus on chromosome 13 was controlled for. The dashed line represents the significant threshold (95%) based on 1000 permutations of the data. Only a single locus reached genome-wide significance, on chromosome 10. (C) Expanded view of a chromosome 10 region showing the broad peak of association with NEU phenotype, which spans 57 genes including the *P*. *falciparum* Merozoite Protein 3 related multigene cluster.
(TIF)

S6 Fig Genome editing strategy targeting both *PfRh2a* and *PFRh2b* genes. A pCC1 vector was designed containing a resistance cassette for hdhfr under the control of the calmodulin promoter flanked by homology regions (HR) found within the sequence shared by both *PfRh2a* and *PfRh2b*. This vector was transfected into 7G8 parasites together with a pDC2 vector containing a CRISPR/Cas9 expression cassette and gRNAs targeting a shared sequence of *PfRh2b* and *PfRh2a*.
(TIFF)

S7 Fig Confirmation by PCR of cassette integration into 7G8 derived clones. Genotyping PCR to confirm cassette integration in the targeted genes. The forward primer hybridises to the cassette while the reverse primers AR1 and AR2 are specific for *PfRh2a* and BR1 and BR2 primers specific for Rh2b (Wt = 7G8, 15B-25F = edited clones of 7G8).
(TIFF)

S8 Fig Confirmation by Illumina sequencing of target deletion in both *PfRh2b* and *PfRh2a* genes in 7G8 derived clones. Coverage plot of mapped Illumina reads in the parental strain 7G8 and Gb4 and two 7G8 derived clones (15D and 15E). A deep decrease in coverage is detected in both genes for the 7G8 derived clones and corresponds to the region target for deletions (red arrows), with 362bp.
(JPG)
