# Supplementary figures and images for: A forward genetic screen reveals a primary role for Plasmodium falciparum Reticulocyte Binding Protein Homologue 2a and 2b in determining alternative erythrocyte invasion pathways
Source: PLoS Pathog. 2018 Nov 29;14(11):e1007436. doi: 10.1371/journal.ppat.1007436 (PMC6289454; doi:10.1371/journal.ppat.1007436)

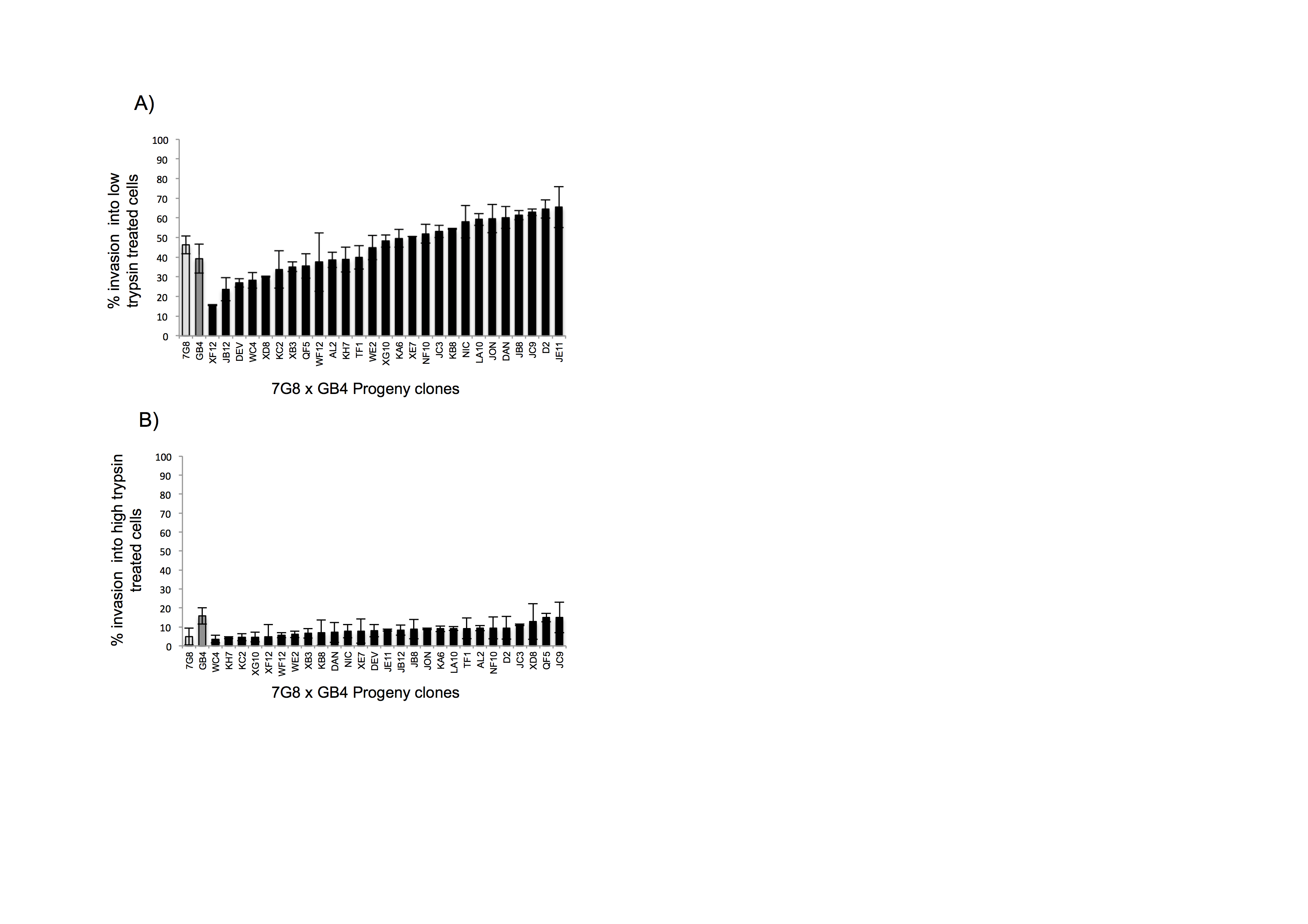

Supplement: S1 Fig — Invasion profiles of 27 progeny clones (black bars) and parental strains (light grey: 7G8, dark grey: GB4) into erythrocytes treated with (A) low trypsin (50 μg/mL) or (B) high trypsin (1 mg/mL). Percentage values are relative to invasion into untreated cells. Results represent mean values from a minimum of 3 biological and 3 technical replicates. (JPG) [file ppat.1007436.s001.jpg]

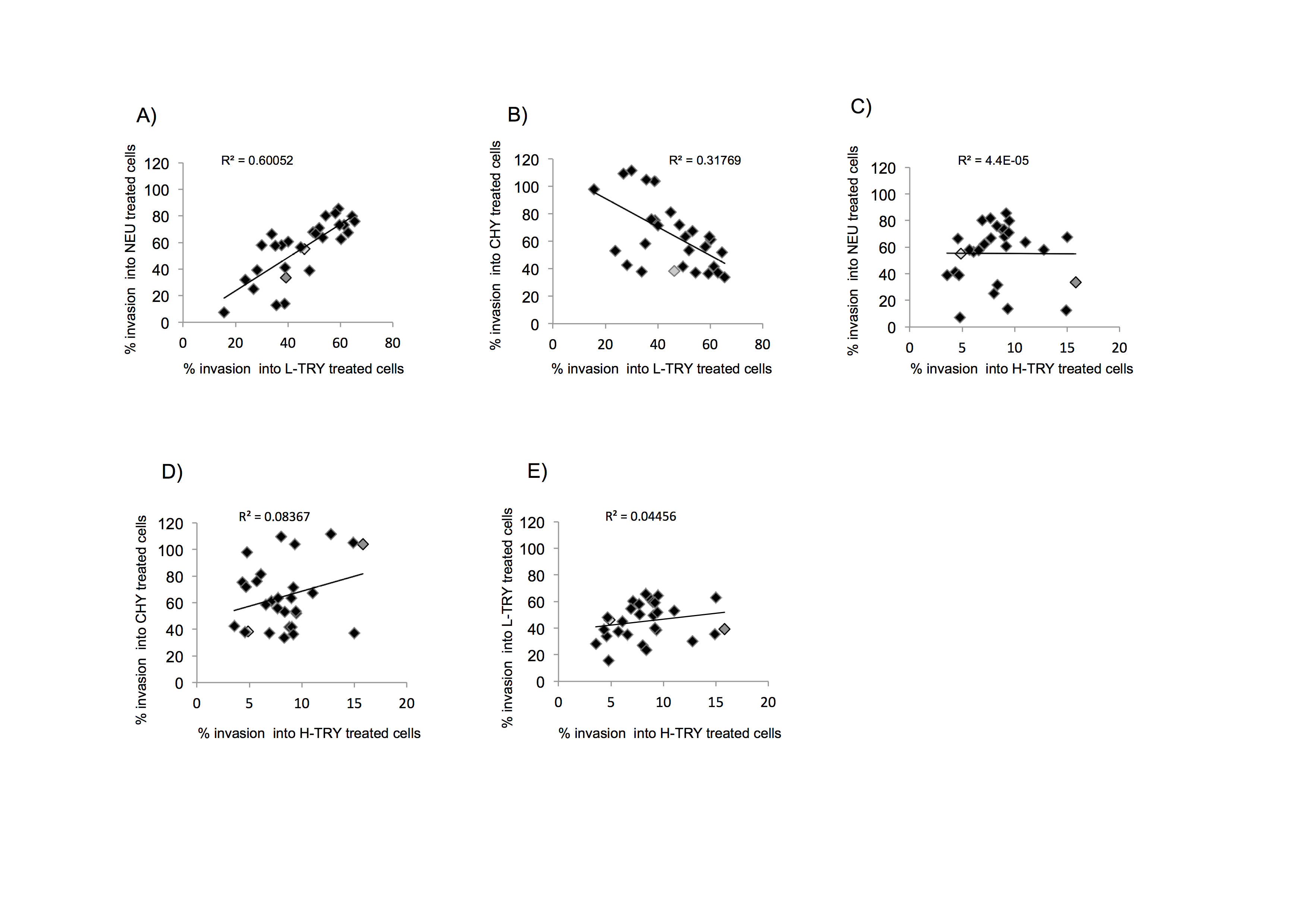

Supplement: S2 Fig — Correlation of mean invasion rates of progeny (black diamond) and parental (7G8: light gray, GB4: dark gray) into enzyme treated erythrocytes. All phenotypes were compared in a pair-wise manner: NEU/L-TRY (A), CHY/L-TRY (B), NEU-H-TRY (C), CHY-H-TRY (D) and L-TRY-H-TRY (E). (JPG) [file ppat.1007436.s002.jpg]

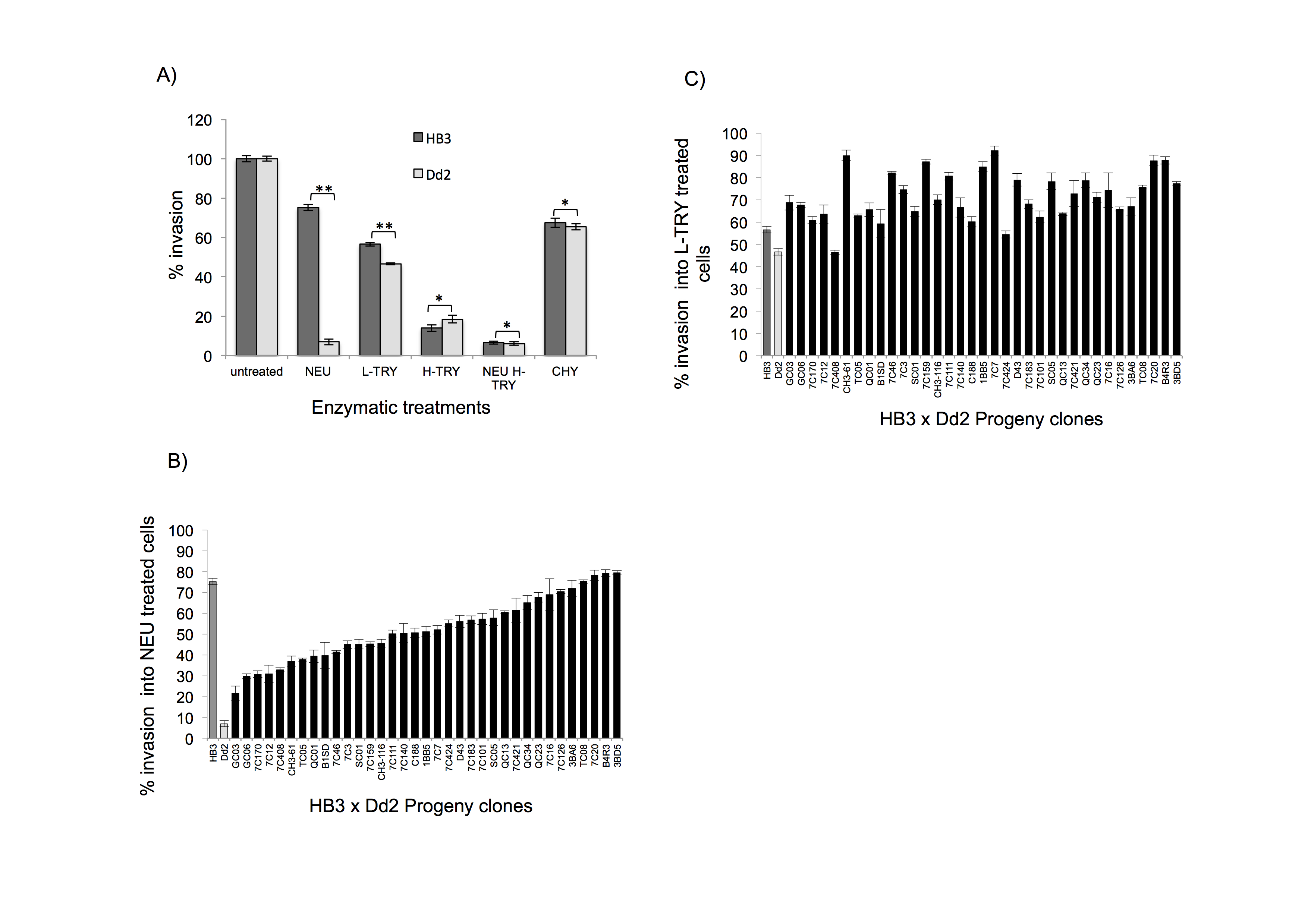

Supplement: S3 Fig — (A) Invasion rates of the Dd2 (light grey bars) and HB3 (dark grey bars) parental strains into enzymatic treated and untreated cells. *p-value> 0.06, ** p-value< 0.00001. (B) Invasion profiles of 35 progeny clones (black bars) and parental strains into NEU (B) and Low(L)-TRY treated (C) cells. Percentage values are relative to invasion into untreated cells. Results represent a minimum of 2 biological and 3 technical replicates. Error bars are standard error of the mean. (JPG) [file ppat.1007436.s003.jpg]

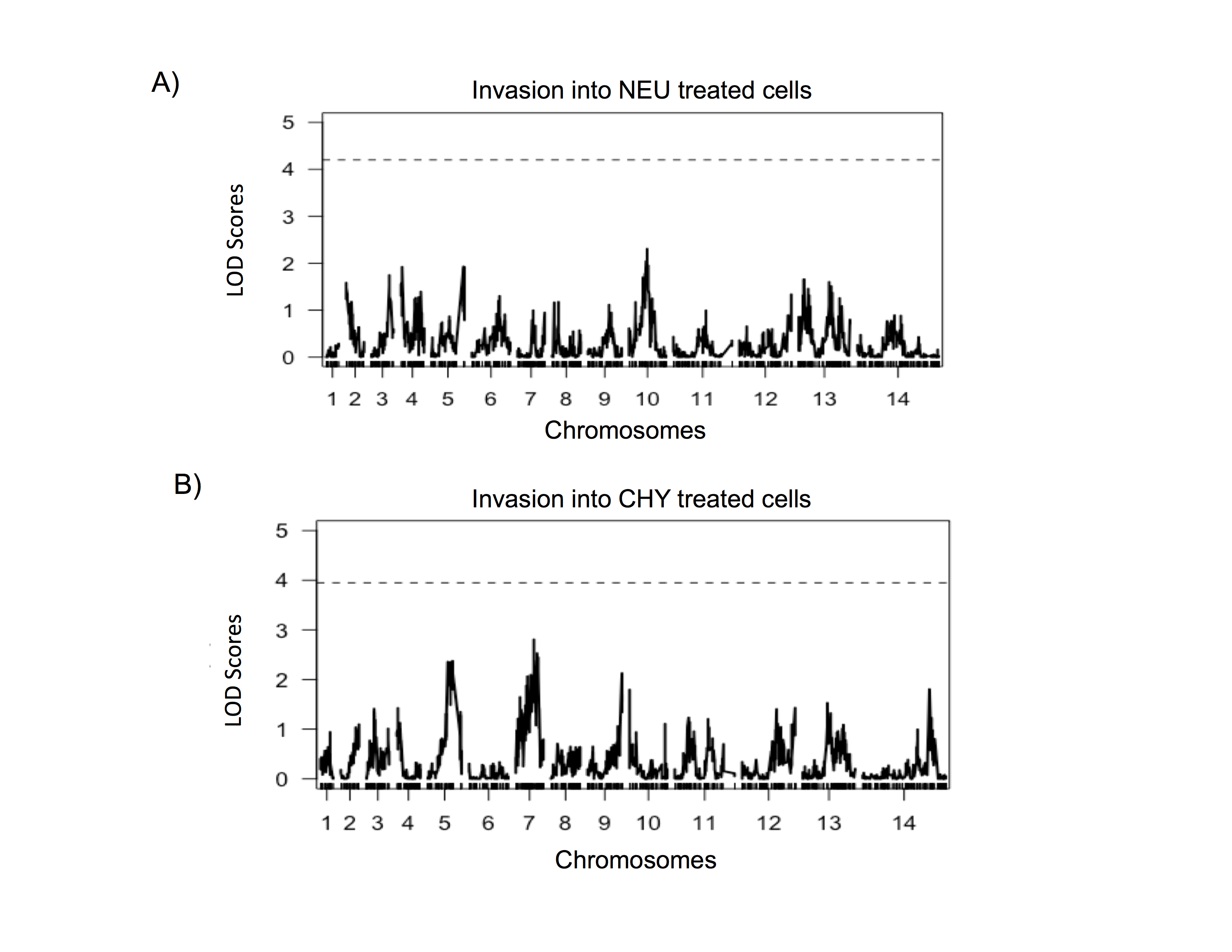

Supplement: S4 Fig — Logarithm of odds (LOD) score results for (A) NEU and (B) CHY invasion phenotypes, correlating with 5,433 SNPs across the genome generated by whole genome sequencing data. The dashed line represents the significant threshold (95%) based on 1000 permutations of the data. No loci reached genome wide significance. (JPEG) [file ppat.1007436.s004.jpeg]

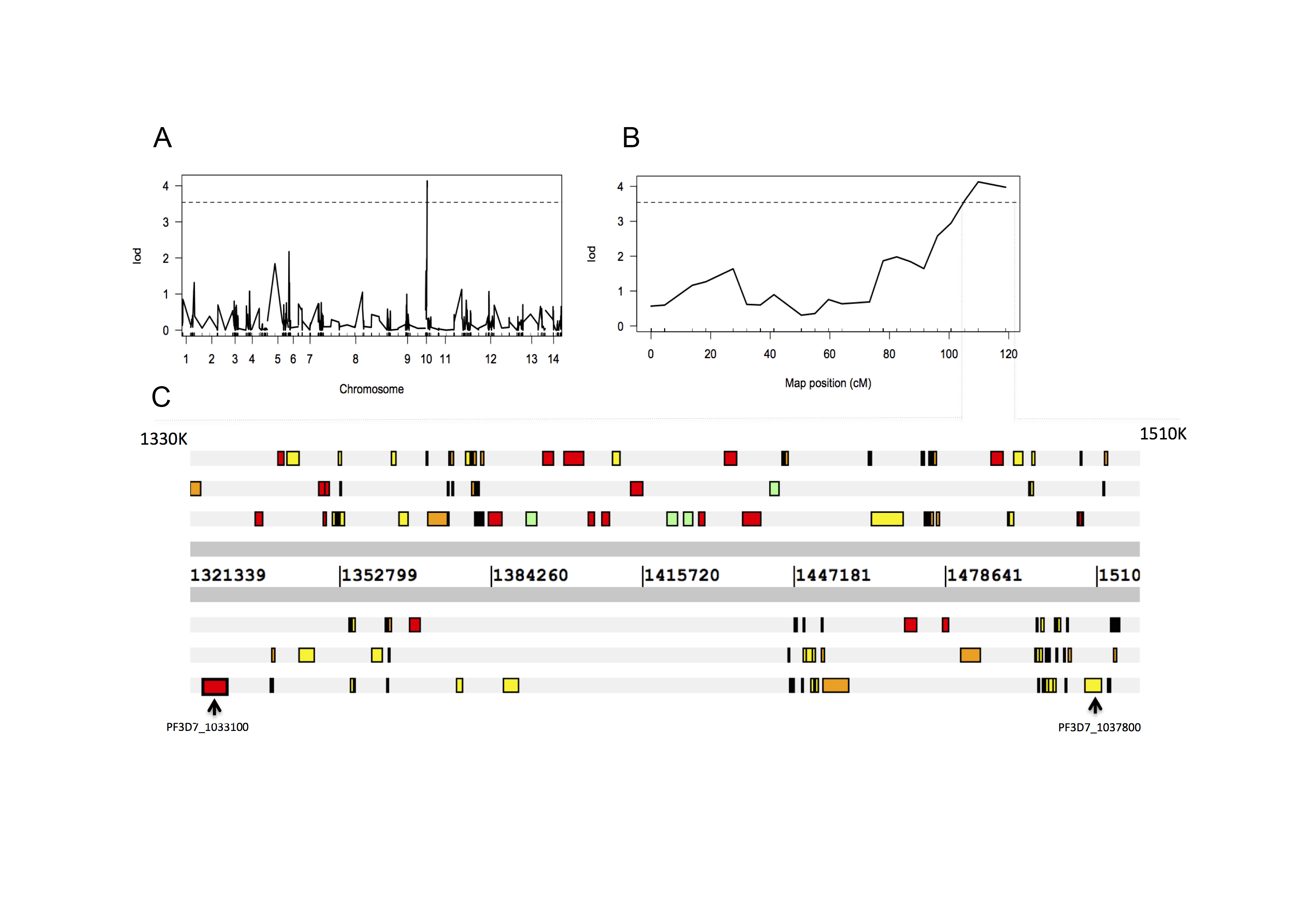

Supplement: S5 Fig — Logarithm of odds (LOD) score results for the invasion phenotype into NEU-treated (A) and CHY-treated (B) cells correlated with 5,433 SNPs across the genome generated by whole genome sequencing data, after variation at the major locus on chromosome 13 was controlled for. The dashed line represents the significant threshold (95%) based on 1000 permutations of the data. Only a single locus reached genome-wide significance, on chromosome 10. (C) Expanded view of a chromosome 10 region showing the broad peak of association with NEU phenotype, which spans 57 genes including the P. falciparum Merozoite Protein 3 related multigene cluster. (TIF) [file ppat.1007436.s005.tif]

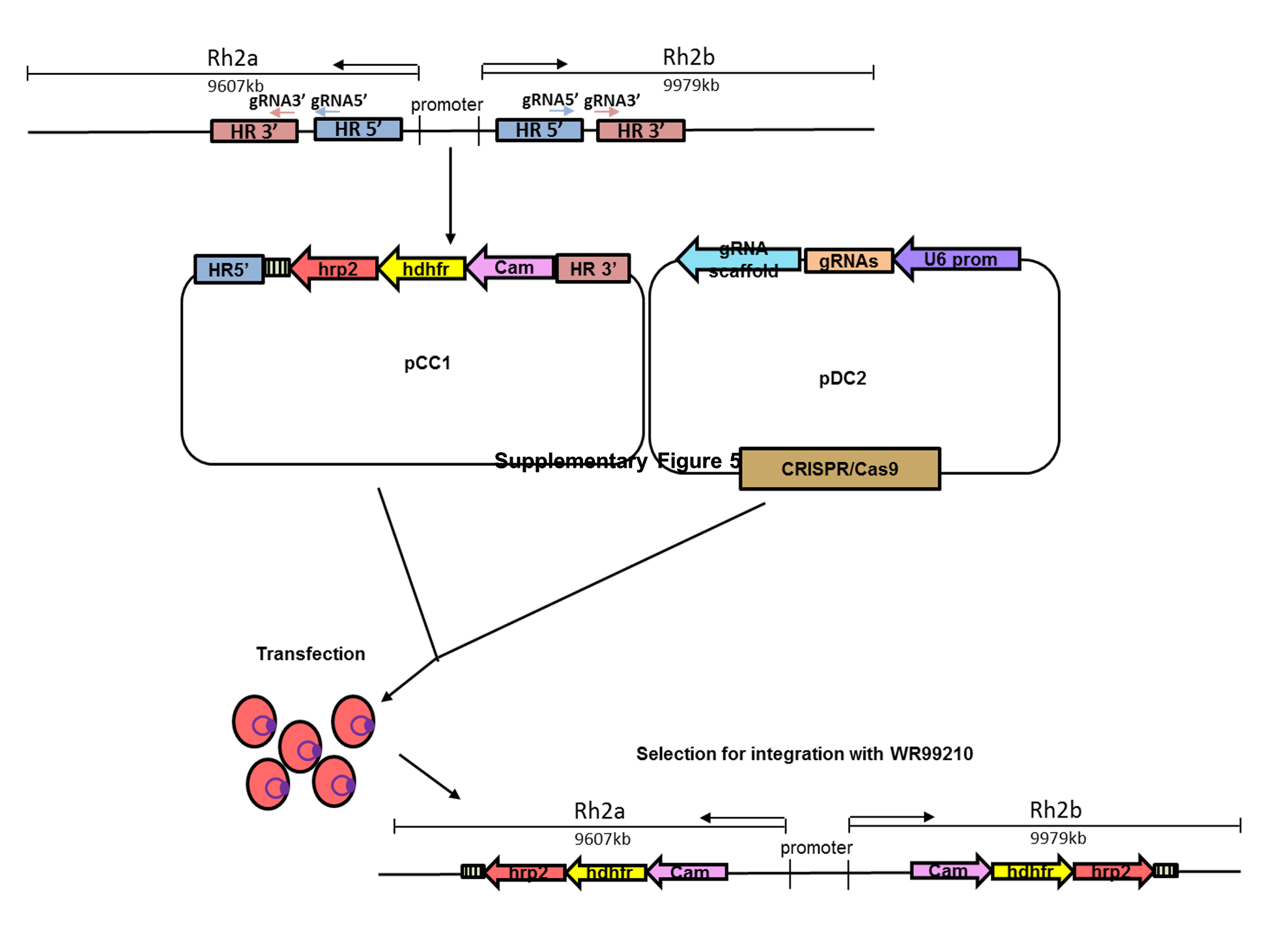

Supplement: S6 Fig — A pCC1 vector was designed containing a resistance cassette for hdhfr under the control of the calmodulin promoter flanked by homology regions (HR) found within the sequence shared by both PfRh2a and PfRh2b. This vector was transfected into 7G8 parasites together with a pDC2 vector containing a CRISPR/Cas9 expression cassette and gRNAs targeting a shared sequence of PfRh2b and PfRh2a. (TIFF) [file ppat.1007436.s006.tiff]

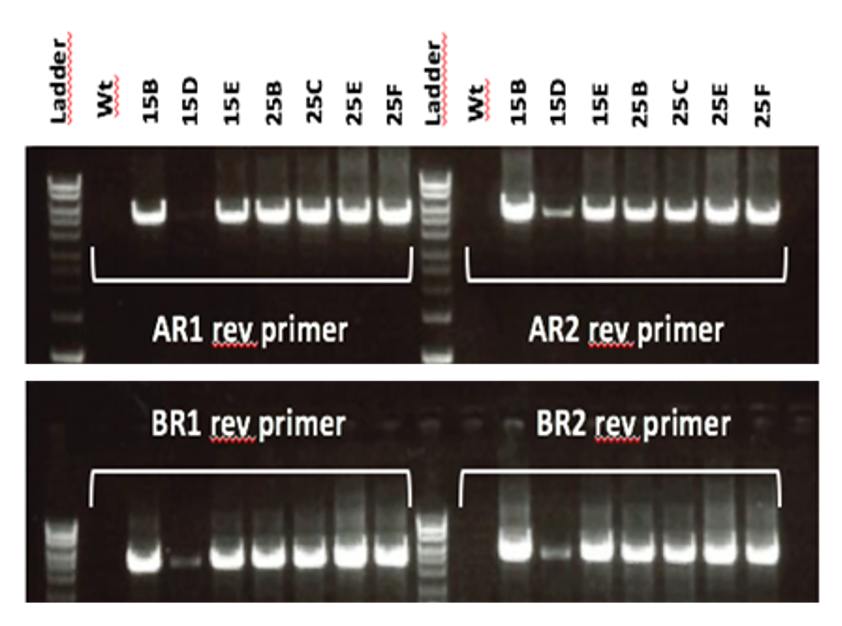

Supplement: S7 Fig — Genotyping PCR to confirm cassette integration in the targeted genes. The forward primer hybridises to the cassette while the reverse primers AR1 and AR2 are specific for PfRh2a and BR1 and BR2 primers specific for Rh2b (Wt = 7G8, 15B-25F = edited clones of 7G8). (TIFF) [file ppat.1007436.s007.tiff]

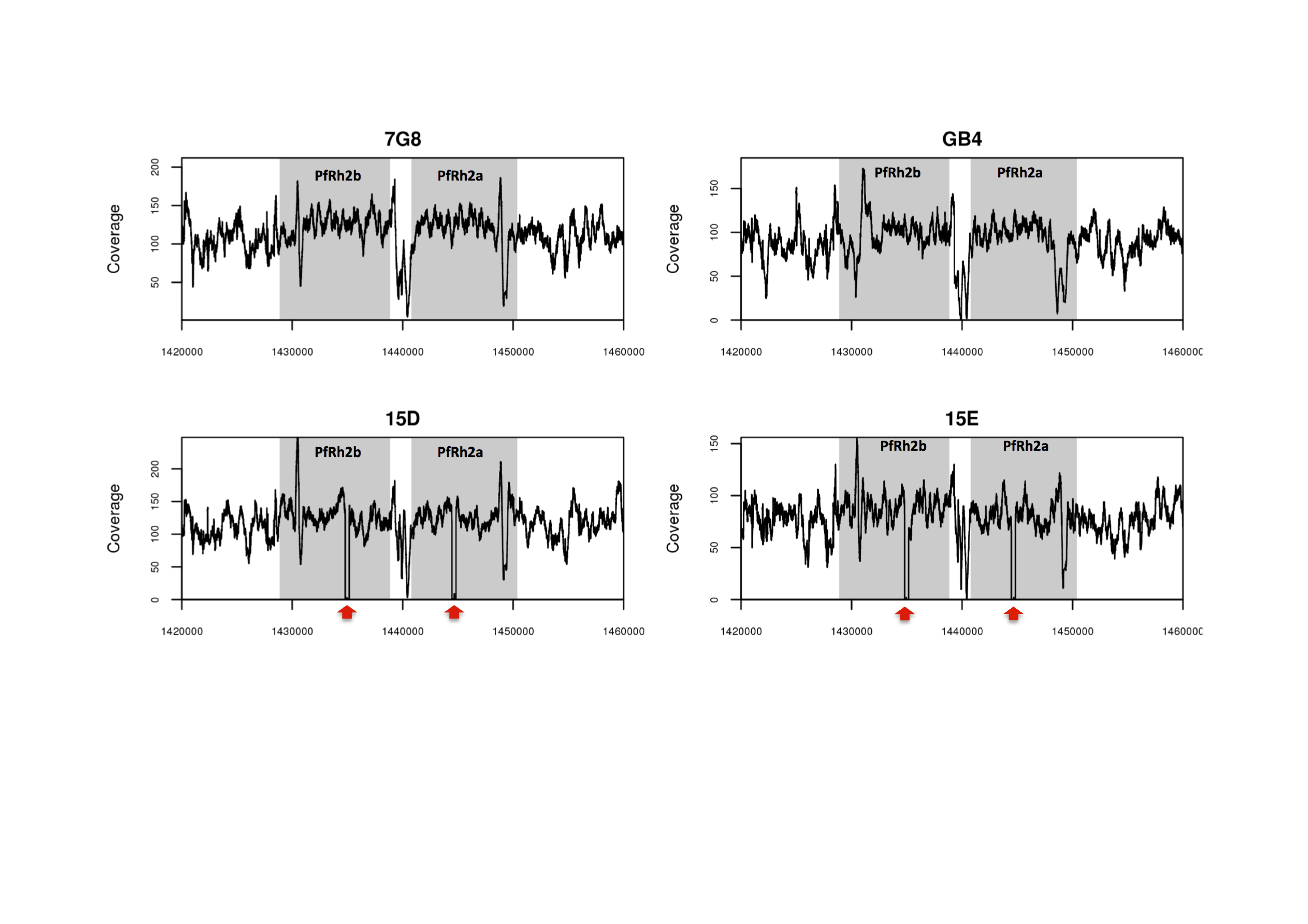

Supplement: S8 Fig — Coverage plot of mapped Illumina reads in the parental strain 7G8 and Gb4 and two 7G8 derived clones (15D and 15E). A deep decrease in coverage is detected in both genes for the 7G8 derived clones and corresponds to the region target for deletions (red arrows), with 362bp. (JPG) [file ppat.1007436.s008.jpg]
